# Virtual Reality Is Sexist: But It Does Not Have to Be

**DOI:** 10.3389/frobt.2020.00004

**Published:** 2020-01-31

**Authors:** Kay Stanney, Cali Fidopiastis, Linda Foster

**Affiliations:** ^1^Design Interactive, Inc., Orlando, FL, United States; ^2^Lockheed Martin Corporate, Washington, DC, United States

**Keywords:** virtual reality, cybersickness, gender differences, interpupillary distance, head mounted displays, motion sickness

## Abstract

The aim of this study was to assess what drives gender-based differences in the experience of cybersickness within virtual environments. In general, those who have studied cybersickness (i.e., motion sickness associated with virtual reality [VR] exposure), oftentimes report that females are more susceptible than males. As there are many individual factors that could contribute to gender differences, understanding the biggest drivers could help point to solutions. Two experiments were conducted in which males and females were exposed for 20 min to a virtual rollercoaster. In the first experiment, individual factors that may contribute to cybersickness were assessed via self-report, body measurements, and surveys. Cybersickness was measured via the simulator sickness questionnaire and physiological sensor data. Interpupillary distance (IPD) non-fit was found to be the primary driver of gender differences in cybersickness, with motion sickness susceptibility identified as a secondary driver. Females whose IPD could not be properly fit to the VR headset and had a high motion sickness history suffered the most cybersickness and did not fully recover within 1 h post exposure. A follow-on experiment demonstrated that when females could properly fit their IPD to the VR headset, they experienced cybersickness in a manner similar to males, with high cybersickness immediately upon cessation of VR exposure but recovery within 1 h post exposure. Taken together, the results suggest that gender differences in cybersickness may be largely contingent on whether or not the VR display can be fit to the IPD of the user; with a substantially greater proportion of females unable to achieve a good fit. VR displays may need to be redesigned to have a wider IPD adjustable range in order to reduce cybersickness rates, especially among females.

## Introduction

In general, those who have studied cybersickness (i.e., the motion sickness associated with VR exposure) and other forms of motion sickness oftentimes report that females are more susceptible than males. Cooper et al. (at sea; 1997), Kaplan (on trains; 1964), Lawther and Griffin (at sea; 1986; sea, 1988), Lederer and Kidera (on planes; 1954), Lentz and Collins (general susceptibility; 1977), Munafo et al. (in VR; 2017); Park and Hu (in a rotating drum; 1999), Stanney et al. (in VR; 2003); Turner and Griffin (in automobiles; 1999), and Turner et al. (on planes; 2000) all found females more susceptible to motion sickness as compared to males across diverse motion platforms. Yet when Lawson (2014) reviewed 46 studies examining gender differences in motion sickness, he reported that only 26/46 (56.5%) found higher levels of susceptibility in females as compared to males. Further, in immersive environments, there are many individual factors that could contribute to gender differences, including previous experience with virtual motion, field of view (FOV), IPD, field dependence, postural stability, female hormonal cycle, state/trait anxiety, migraine susceptibility, ethnicity, aerobic fitness, body mass index, among others (see Tables 1, 2). Researchers have yet to identify which of these factors are the primary drivers of susceptibility differences between the genders. Of the studies that do exist, generally only a few variables were considered at one time rather than examining across a large number of potential drivers (c.f. Parkman et al., 1996; Stanney et al., 2003; Klosterhalfen et al., 2005). In addition, gender differences in susceptibility have been speculated to be attributed to differences in symptom awareness and willingness to report symptomatology. However, past studies have shown a 5:3 female to male risk ratio for vomiting, which is an objective measure of motion sickness (Lawther and Griffin, 1986). Examining differences from a physiological level can address any such reporting differences. Yet, even from a physiological level conflicting data exist. While females have been shown to have higher emetic response rates (Kennedy et al., 1995; Golding, 2006), as well as greater sensitivity in peripheral alpha- and beta- adrenergic receptors (Girdler et al., 1990; Kajantie and Phillips, 2006), which increases autonomic responses associated with motion sickness (Finley et al., 2004), Jokerst et al. (1999) found no significant differences between the genders in gastric tachyarrhythmia during exposure to an optokinetic drum, and Cheung and Hofer (2002) found no significant gender-based physiological differences during coriolis cross-coupling stimulation. Thus, while females are generally thought to have higher susceptibility to cybersickness than males, this relationship has not been well-characterized, especially for the latest generation of VR headsets.

**Table 1:** Traits and factors that may affect cybersickness susceptibility in females.

**Traits and factors**	**Gender difference**	**Impact**
Underlying physiological mechanisms	Females tend to exhibit greater effects of Neurokinin-1 (NK-1) signaling (receptor involved in nausea; Arslanian-Engoren and Engoren, 2010), have higher activation of the limbic system (involved in the generation of nausea; Wang et al., 2007), and exhibit different gastric dysrhythmia based on menstrual cycle phase (Parkman et al., 1996) than males.	Females may be physiologically “hard-wired” for motion sickness susceptibility (Golding, 2006), which can be upregulated via hormonal fluctuations.
Previous experience	In U.S., 59% of males and 41% of females self-report as gamers [Entertainment Software Association (ESA), 2016]. Yet, hardcore gamers who play >5 h per week remain primarily male (NPD Group, 2014) and early adopters of VR technology are primarily hardcore gamers (Leibach, 2015). Thus, one can speculate that to date, those who have experienced VR are mostly male.	If females have less VR experience than males, it may predispose them to cybersickness as motion sickness is postulated to be due to sensory conflicts between expected patterns of afferent signals (reafference) established through previous experiences and what is being experienced in a novel and sensorially altered environment (Reason and Brand, 1975; Oman, 1998).
Field of view	Females have slightly larger peripheral vision fields (Burg, 1966), slightly higher vertical field of view (Williams and Thirer, 1975), and more active dorsal visual stream and thus better peripheral vision (Becker-Bense et al., 2012; Amen et al., 2017) than males (Lawson, 2014).	If females have a wider FOV and are more sensitive to the peripheral color pallet than males, this may drive higher levels of vection, which in turn may drive higher levels of cybersickness (Webb and Griffin, 2003; Diels and Howarth, 2013).
Interpupillary distance (IPD)	IPD (the distance between the pupils of both eyes) has been found to vary by gender (Fledelius and Stubgaard, 1986; Gordon et al., 2014), with adult females ranging from an IPD of 51–74.5 mm, with a mean of 61.7 mm, and adult males ranging from an IPD of 53–77.5 mm, with a mean of 64 mm. When one matches these IPD ranges to the IPD ranges supported by current VR headsets, it becomes evident that some of today's VR headsets may not fit upwards of 30% or more of females (see Table 2).	IPD range facilitates the correct positioning of VR headset lenses, as there are specific points on the lenses which have to coincide with the center of the pupil (visual axis) of each eye in order for the display image to be in focus. If a VR headset does not allow for such eye-lens alignment, which is much more likely in females (Fulvio et al., 2018), eyestrain and headaches can be expected (Ames et al., 2005), as well as incorrect perception of displayed imagery (Priot et al., 2006).
Field dependence	Gender-based differences have been found in field dependence (FD), perception of veridical vertical with body tilt, perception of the morphological horizon, and mental rotation ability, with males generally far out-performing females (order of one standard deviation higher; Witkin and Goodenough, 1977; Harris, 1978; Darlington and Smith, 1998; Parsons et al., 2004).	If females are more likely to be FD and have difficulty with visuo-spatial tasks than males, this may predispose them to higher motion sickness susceptibility (Parker and Harm, 1992).
Postural stability	The spatial magnitude of postural sway and the control of posture differs between genders, with females demonstrating more multifractality of postural sway (Koslucher et al., 2016).	If females are less able to control and stabilize their bodily activity than males, this may predispose them to higher motion sickness susceptibility according to the ecological theory (Riccio and Stoffregen, 1991).
Female hormonal cycle	Motion sickness susceptibility fluctuates throughout the menstrual cycle, with this fluctuation in susceptibility across the cycle accounting for approximately one-third of the overall difference between the genders in motion sickness susceptibility (Golding et al., 2005).	If females are more susceptible to cybersickness during certain phases of the hormonal cycle, this may render them less capable of tolerating VR exposure as compared to males during these peaks.
State and trait anxiety	Females report higher trait-anxiety (Robin et al., 1987), with incidence in females >2x as high as males (Donner and Lowry, 2013), which may in-turn drive increased cortisol levels (Meissner et al., 2009), and affect neuronal activity within the amygdala (Sandi et al., 2008), with phasic activation in the amygdala being shown to precede strong nausea (Cha et al., 2012; Napadow et al., 2013). State anxiety may drive disorientation and vertigo (Brandt, 1996), which can drive motion sickness.	Heightened anxiety in females may render them more susceptible to motion sickness than males, as heightened state- (Tucker and Reinhardt, 1967) and trait- anxiety (Paillard et al., 2013) are strongly related to cybersickness (Ling et al., 2011).
Migraine susceptibility	Females are susceptible to migraines, ~3x more prone to than males (Rasmussen et al., 1991), and migraine sufferers have a global hypersensitivity to different sensory stimuli (Granziera et al., 2006), anomalies in early motion processing pathways underlying contrast sensitivity (Singh and Shepherd, 2016), and vestibular abnormalities (e.g., vestibulo-ocular reflex dysfunction; Kim et al., 2005), which may contribute to spatial disorientation (Cho et al., 1995).	If females are more predisposed to migraines and associated vestibular abnormalities than males, this may predispose them to higher motion sickness susceptibility (Golding, 1998; Marcus et al., 2005).
Ethnicity	Genetic factors account for ~half of variation in motion sickness susceptibility (Reavley et al., 2006), with Asians being more susceptible than African Americans (Stern et al., 1993) and Caucasians (Klosterhalfen et al., 2005). The “gg” phenotype is 5.8x more common in Chinese than in European Caucasians, as well as 1.6x more common in those susceptible to motion sickness (Liu et al., 2002).	When comparing differential effects of ethnicity and gender, ethnicity may be the strongest intrinsic factor contributing to motion sickness, with gender playing a more modest role (Klosterhalfen et al., 2006); or there may be an interaction effect (Stern et al., 1993).
Body mass index (BMI)	Higher BMI may somewhat moderate motion sickness (Stanney et al., 2003; Yi et al., 2017), as adiposity may be protective against emetic responses in that it is associated with diminished activity of the gastrointestinal system (Kohl, 1990).	If females have a higher proportion of adipose tissue as compared to males (Hellstroèm et al., 2000), this difference may lead to males being more susceptibility to motion sickness than females.
Aerobic fitness	Males have been shown to have higher aerobic capacity than females with comparable training (Sandbakk et al., 2012), which has also been associated with increased motion sickness susceptibility (Banta et al., 1987). Rawat et al. (2002) suggested that vasomotor susceptibility (e.g., epigastric discomfort, nausea, vomiting, headache) may be higher in aerobically fit individuals susceptible to motion sickness. Yet, Jennings et al. (1988) found no such association.	If females have less aerobic capacity than males, this difference may lead to males being more susceptibility to motion sickness than females.
Past motion sickness history	Females are generally more inclined to be aware of and admit subjective symptoms, as well as more likely to remember past motion sickness experiences as compared to males (Jokerst et al., 1999; Park and Hu, 1999; Cheung and Hofer, 2002; Flanagan et al., 2005; Golding et al., 2005). Dobie (1974) found little evidence that men are more reticent to report motion sickness as compared to females.	If females are more inclined to report and be aware of motion sickness symptomatology as compared to males, this could lead to an overestimation of gender differences that are not corroborated via physiological assessment.

**Table 2:** IPD gender range vs. current virtual reality headsets.

**Headset**	**IPD range**
Sony PlayStation	Adjustable via software between 48 and 78 mm (VR Heads, 2017), and would be expected to fit the entire adult population
Samsung Gear VR	Fixed at 62 mm (Samsung, 2016), and would only be expected to fit individuals with an IPD of 62 mm, which is ~10% of both males and females (Gordon et al., 2014)
Oculus Rift	Adjustable between 58 and 72 mm (Carbotte, 2016), and thus would not be expected to fit the smallest ~15% of females and the largest ~1% of both males and females
Oculus Rift S	Adjustable between 61.5 and 65.5 mm (Heaney, 2019), and thus would not be expected to fit the smallest ~45% of females, the largest ~15% of women, the smallest ~20% of males, and the largest ~30% of males
Oculus Quest	Adjustable between 56 and 74 mm (Heaney, 2019), and thus would not be expected to fit the smallest ~7% of females and the largest ~1% of males
HTC Vive	Adjustable from 60.5 to 74.4 mm (HTC Vive, 2017), and thus would not be expected to fit the smallest ~35% of females, smallest ~15% of males, and largest ~1% of males
HTC Vive Pro	Adjustable from 60.9 to 74 mm (HTC Vive Pro, 2018), and thus would not be expected to fit the smallest ~40% of females, smallest ~18% of males, and largest ~1% of males

*Females IPD Range: 51–74.5 mm (mean IPD = 61.7 mm; S.D. = 3.6 mm); Male IPD Range: 53–77.5 mm (mean IPD = 64.0 mm; S.D. = 3.4 mm). Percentages based on US Army Anthropomorphic Survey (ANSUR) database (Gordon et al., 2014)*.

Why do gender differences matter? VR technology is anticipated to fill many enterprise roles in the coming decades, from training to maintenance to operational support to design, and more. Currently >150 companies in multiple industries, including >50 Fortune 500 companies, are testing and/or deploying VR solutions (Kaiser and Schatsky, 2017; Morris, 2018). As VR-based real-time guidance systems driven by artificial intelligence advance, persons who cannot tolerate these delivery systems may be left out of job advancement. We cannot create a divide, with those who can handle VR exposure advancing due to better, more immersive training, more effective repair jobs aided by real-time augmented guidance, more creative designs that evolve from a mesh of digital and physical worlds, etc., while those who are susceptible to cybersickness are left on the sidelines watching this new era of VR empowered productivity pass them by. Further, if the design of VR headsets is discriminative to females, they, in particular, may experience challenges when trying to harness the bevy of performance enhancing potential of VR enterprise applications.

The main goal of this study was to determine what the primary drivers of gender-based differences in cybersickness susceptibility within VR environments are so that potential countermeasures to better accommodate females can be identified. To this end, two experiments were conducted. The first study examined potential drivers of cybersickness, which are summarized in Table 1, to identify those that may be contributing the most to gender differences. It was anticipated that a subset of these factors would be identified as particularly influential in driving higher levels of cybersickness among females.

## Experiment 1

### Materials and Methods

The purpose of Experiment 1 was to determine how well males and females are able to tolerate VR exposure and what factors might be driving any differences in the cybersickness they may experience. Based on the studies summarized in Table 1, it was anticipated that females would experience higher levels of cybersickness than males, with the goal of the experiment being to identify which factors drive any such differences.

#### Participants

Adults aged 18–30 years, balanced between genders participated in this study. Participants were recruited through a market research firm. A total of 46 participants participated in the study and were randomized to either an experimental group (VR headset; *n* = 30 [15 male/15 female]) or a control condition (flatscreen television; *n* = 16 [8 male/8 female]). This research complied with the American Psychological Association Code of Ethics and was approved by the Institutional Review Board at Copernicus Group. Informed consent was obtained from each participant and all participants were compensated for their time in the experiment.

#### Equipment and Display Content

The displays used in this study included the HTC Vive VR headset (which does not fit, on average, ~35% of females and ~16% of males based on the adjustable IPD range) and a flatscreen television. The HTC Vive has OLED display technology, a resolution of 2,160 × 1,200 (1,080 × 1,200 per eye), a refresh rate of 90 Hz, a field of view of 110 degrees, weight of 555 g (1.22 lbs), and an IPD range adjustable from 60.5 to 74.4 mm. The flatscreen television was a Samsung H6350 Smart LED TV with a screen size of 60.0′′ measured diagonally and a resolution of 1,920 × 1,080.

**Figure 1:**
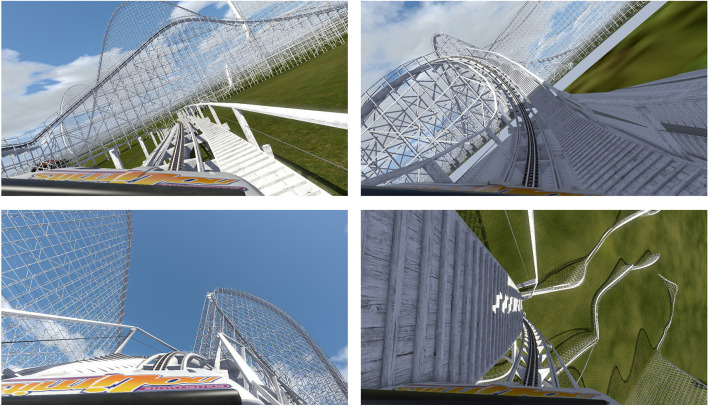
Views from VR rollercoaster.

Steam platform was used to develop a virtual rollercoaster of 20 min duration (see Figure 1). In order to create provocative content that would instigate cybersickness, the following factors were incorporated into the virtual rollercoaster ride:

- Off-vertical axis rotation (visual OVAR; e.g., rollercoaster wraparounds, spinning track), as OVAR can be expected to lead to extreme levels of nauseogenicity (Golding et al., 2009);
- Variable velocity, forward acceleration, and vertical acceleration via humps in the track that provided visual oscillation, as these motions are known to be provocative (Alexander et al., 1947; Lawther and Griffin, 1986);
- High level of optic flow (implemented via movement through support structures, maintenance gangways, ground tunnels, and other visual details), which tends to drive visually induced motion sickness (Smart et al., 2014);
- Anchoring to the lead rollercoaster car with no car in front to focus on, as a fixed-horizon or stable vehicle dashboard reduces cybersickness (Prothero and Parker, 2003);
- Constant, rhythmic, and repetitive sound that simulated movement along the track so that participants were visually and aurally convinced they were moving when they were actually sitting still in a chair, as such sounds can drive nausea and disorientation (Dawson, 1982); and
- No control by the participant over virtual motion, as lack of viewpoint control has been demonstrated to be very nauseogenic (Stanney and Hash, 1998).

To maintain consistency in the visual stimulus across groups, the SteamVR format was exported to a video format to run in flatscreen television format.

#### Procedure

The experiment involved the following phases— pre-screening, screening, pre-testing, immersive exposure, and post-testing.

In the pre-screening phase, a participant recruiter called potential participants and reviewed inclusion requirements with them to identify candidate participants. Any participant reporting affirmative to any exclusion criteria (neurological impairments, musculoskeletal problems of the knee, ankle, shoulder, and/or elbow, loss in depth perception, <20/20 corrected visual acuity, inner-ear anomalies, history of seizures, pregnancy) was not asked to participate in the study. Participants who met pre-screening eligibility and inclusion requirements were scheduled for on-site screening. During the on-site screening: (1) upon arrival, participants were welcomed, and provided with informed consent documentation; (2) all participants were provided with a 3-digit number based on order of participation and experimental condition that was used for data collection; (3) a Simulator Sickness Questionnaire (SSQ; Kennedy et al., 1993) was administered electronically and participants that scored > 12 were thanked for their willingness to participate and excluded from the study; (4) a visual acuity test was administered and participants who did not have corrected 20/20 vision were thanked for their willingness to participate and excluded from the study; and (5) the Titmus Stereotest was administered to assess depth perception and participants that scored <6/9 were thanked for their willingness to participate and excluded from the study. Participants who met screening eligibility proceeded to pre-testing.

During the pre-testing phase, participants completed a demographics form via which they reported their previous VR and gaming experience, phase of the menstrual cycle (female only), and ethnicity, as well as other demographic data. Participants FOV (i.e., open observable area an individual can see through their eyes, which covers both central foveal and peripheral vision; Strasburger et al., 2011) was then measured via a vision protractor, their IPD (i.e., distance between the center of their pupils; Dodgson, 2004) was measured via a digital pupilometer (binocular pupillary range: 45–80 mm), their weight and height were measured to assess body mass index, their aerobic fitness (i.e., peak expiratory flow) was assessed via the Philips Respironics HS755 Personal Best Full Peak Flow Meter, and postural stability was assessed via the Sharpened Rhomberg Test (Johnson et al., 2005) using a Polhemus G4 wireless magnetic motion-tracking device with the sensor mounted via a naval strap. Participants then filled out surveys, including the State–Trait Anxiety Inventory (STAI; Spielberger et al., 1970), Motion History Questionnaire (MHQ; Kennedy et al., 1992), Cube Comparison Survey (Ekstrom et al., 1976), and Migraine Susceptibility Survey based on the International Headache Society [International Headache Society (IHS), 2017] Criteria for Diagnosing Migraine.

During the immersive exposure phase, participants were randomized to a control (i.e., flatscreen television) or experimental group (i.e., VR headset) and fitted with physiological sensors of electrocardiogram (ECG; to assess alterations to cardiovascular activity, i.e., heart rate), electrogastrography (EGG; to assess abnormal gastric rhythms, including tachygastria and bradygastria), and electrodermal activity (EDA; to assess skin conductance level [SCL]). Following a 5 min baselining of the physiological measures, participants were exposed to immersive content (virtual rollercoaster) for 20 min. The IPD of participants in the VR group was entered into the headset software and adjusted on the headset prior to viewing the rollercoaster stimuli to the best match available based on the IPD range of the HTC Vive. Those participants with an IPD smaller or larger than the HTC VIVE range were, respectively, given the value at the lowest or highest value available (60.5 or 74.4 mm). Participants were monitored via the physiological measures throughout VR exposure.

During the post-testing phase, the SSQ Total Score was assessed immediately following the immersive exposure (AE [aftereffects] 1), and in 15 min increments for a total of 60 min (AE2–AE5) post exposure. Participants were then debriefed, thanked, and paid for participation.

#### Experimental Design

The experiment was a mixed design, with 2 (gender) × 2 (display type) between factors and a 5 (post exposure measurement time) within factor. The display types were VR headset and flatscreen television and gender types were male and female. The post exposure measurement times were 0, 15, 30, 45, and 60 min.

#### Predictor Variables

The cybersickness predictor variables (see Table 3) included self-report measures (previous VR and gaming experience, female hormonal cycle, ethnicity—all gathered via demographics form), survey assessed measures (field dependence, state/trait anxiety, migraine susceptibility, past motion sickness history), and body measures (FOV, IPD, postural stability, aerobic fitness, body mass index, ECG, EGG, EDA).

**Table 3:** Predictor variables investigated.

**Variable**	**Description**	Experiment 1 inclusion in regression analysis^*^	Experiment 2 inclusion in regression analysis^*^
Gender	Participant gender; Coded as Male = 0; Female = 1	Included in model	Included in model
Physiological mechanisms	Measurement of EGG, heart rate, and skin conductance level during exposure	Not included in model, no significant differences in EGG, heart rate, and skin conductance level between genders	Included EGG (bradygastria) in model; on average females had higher levels of bradygastria than males (female mean = 29.72 [S.D.= 2.47]; male mean = 19.17 [S.D. = 2.16], *F* = 10.37, *p* = 0.002)
Previous experience	A demographics questionnaire was used to gather previous experience VR and gaming	Included in model; on average, females had less previous VR (female mean = 1.24 h [S.D. = 3.16]; male mean = 8.36 h [S.D. = 24.85]; *F* = 3.22; *p* = 0.077) and gaming (female mean = 6.63 h [S.D. = 10.13]; male mean = 12.11 h [S.D. = 14.16]; *F* = 3.97; *p* = 0.056) experience than males	Included in model; on average, females had less previous VR experience (female mean = 4.33 h [S.D. = 12.77]; male mean = 7.11 h [S.D. = 19.03]; *F* = 0.848; *p* = 0.359 and gaming (female mean = 7.03 h [S.D. = 14.11]; male mean = 12.53 h [S.D. = 10.77]; *F* = 5.03; *p* = 0.023) than males
FOV	Participant field of view	Included in model; on average, males had significantly wider FOV (female mean = 152.63 degrees [S.D. = 14.37]; male mean = 158.82 [S.D. = 11.12]; *F* = 3.97; *p* = 0.039) than females	Included in model; on average, males had wider FOV (female mean = 155.25 degrees [S.D. = 32.12]; male mean = 160.52 [S.D. = 12.79]; *F* = 1.23; *p* = 0.270) than females
IPD Fit	Measurement of how well the interpupillary distance could be matched to the worn VR device; Coded as IPD Fit = 1; IPD Non-Fit = 2	Included in model; on average, males had significantly wider IPD (female mean = 62.63 mm [S.D. = 3.52]; male mean = 65.33 mm [S.D. = 2.99]; *F* = 5.12; *p* = 0.031) than females; 5/15 females had an IPD non-fit; while no males had an IPD non-fit	Included in model; on average, males had significantly wider IPD (female mean = 60.80 mm [S.D. = 3.92]; male mean = 62.88 mm [S.D. = 4.37]; *F* = 7.12; *p* = 0.001) than females
Field dependence	Measured via cube comparison; Coded as field dependent = 1; Field intermediate = 2; Field independent = 3	Included in model; on average, males had higher field independence (female mean = 0.56 [S.D. = 10.87]; male mean = 6.33 [S.D. = 14.65]; *F* = 2.99; *p* = 0.088) than females	Not included in model; no significance between genders (female mean = 1.92 [S.D. = 0.836]; male mean = 1.95 [S.D. = 0.80]; *F* = 0.047; *p* = 0.83) than females
Postural stability	Measurement of pre exposure postural stability	Not included; no significance between genders (female mean = 0.527 [S.D. = 1.601]; male mean = 0.671 [S.D. = 1.535]; *F* = 0.452; *p* = 0.50)	Not included; no significance between genders (female mean = 4.99 [S.D. = 8.76]; male mean = 3.57 [S.D. = 3.87]; *F* = 1.089; *p* = 0.299)
Female hormonal cycle	Report of stage in hormonal cycle; Coded as: Male-0; Premenstrual-1; Menstruation-2; Postmenstrual-3; Ovulation-4; Menopause- 5	Included in model	Included in model
State anxiety	Measured by the STAI	State anxiety included in model; on average, males had higher state anxiety pre-exposure (female mean = 26.82 [S.D. = 7.01]; male mean = 31.27 [S.D. = 10.19]; *F* = 4.67; *p* = 0.03) than females	State anxiety included in model; on average, females had significantly higher state anxiety pre-exposure (female mean = 26.55 [S.D. = 6.26]; male mean = 25.81 [S.D. = 5.83]; *F* = 0.44; *p* = 0.51) than females
Trait anxiety	Measured by the STAI	Not included; no significance between genders (female mean = 31.84 [S.D. = 10.18]; male mean = 33.63 [S.D. = 8.67]; *F* = 0.837; *p* = 0.36)	Not included; no significance between genders (female mean = 31.15 [S.D. = 10.19]; male mean = 29.96 [S.D. = 7.24]; *F* = 0.497; *p* = 0.482)
Migraine susceptibility	Indicates an individual's susceptibility to migraine; Coded as migraine non-susceptible = 0; Migraine susceptible = 1	Not included; no significance between genders (females- 13/15 not migraine sufferers; males- 15/15 not migraine sufferers; *F* = 0.11; *p* = 0.74)	Not included; no significance between genders (females- 48/60 not migraine sufferers; males- 48/58 not migraine sufferers)
Past motion sickness history	Measured via MHQ, which assess an individual's exposure to various forms of motion and the occurrence of illness associated with such motion	Included in model; on average, females had higher past motion sickness history (female mean = 3.21 [S.D. = 2.43]; male mean = 2.47 [S.D. = 1.87]; *F* = 1.91; *p* = 0.17) than males	Included in model; on average, females had significantly higher past motion sickness history (female mean = 3.32 [S.D. = 2.94]; male mean = 2.30 [S.D. = 1.99]; *F* = 4.49; *p* = 0.036) than males
Ethnicity	Sample size per group was too low resulting in low power, so non-Caucasian was collapsed and coded as non-Caucasian = 0; Caucasian = 1	Included in model; Participant pool was ~16% African American, ~4% Asian, ~55% Caucasian, ~14% Hispanic, and ~11% of multiple ethnicities	Included in model; Participant pool was ~6% African American, ~4% Asian, ~69% Caucasian, ~6% Hispanic, ~2% American Indian or Alaskan Native, and ~13% of multiple ethnicities
Aerobic fitness	Measured via peak expiratory flow. Coded as low = 0; normal = 1; high = 2	Included in model; males had higher aerobic capacity (female mean = 290.19 [S.D. = 78.40]; male mean = 378.44 [S.D. = 99.37]; *F* = 13.85; *p* < 0.0001) than females	Included in model; males had higher aerobic capacity (female mean = 329.58 [S.D. = 79.91]; male mean = 467.40 [S.D.= 110.23]; *F* = 58.41; *p* = 0.001) than females
Body mass index	Ratio of an individual's height vs. weight	Not included; no significance between genders (female mean = 27.0 [S.D. = 8.56]; male mean = 27.0 [S.D. = 7.21]; *F* = 0.039; *p* = 0.84)	Not included; no significance between genders (female mean = 25 [S.D. = 5.40]; male mean = 26 [S.D. = 6.62]; *F* = 0.281; *p* = 0.597)
VR exposure duration	Length of time participant spent in VR rollercoaster	Not included	Included in model; males had higher exposure duration (female mean = 18.31 [S.D. = 4.17]; male mean = 19.48 [S.D. = 1.96]; *F* = 3.73; *p* = 0.056) than females

* *p < 0.27 level (Bursac et al., 2008)*.

#### Dependent Measure

The dependent measure was cybersickness as measured by the SSQ Total Score (TS; Kennedy et al., 1993) at 0, 15, 30, 45, and 60 min post exposure. The time component after VR exposure is critical to understanding the sustained negative effects of exposure on an individual (Stanney and Hash, 1998). Thus, for the purposes of regression analysis, cybersickness was operationalized as a “recovery” SSQ Total Score (TS), which was defined by the average SSQ TS 45 min post exposure and SSQ TS 1 h post exposure normalized by the Baseline (BL). Given a 20 min VR exposure duration and 1 h post exposure measurement period (i.e., 3x exposure duration), participants would be expected to have “recovered” to BL SSQ TS levels at the conclusion of the experiment.

#### Data Analysis

A mixed-model analysis of variance (ANOVA) was used to identify main and interaction effects among Gender, Display Type, and Post Exposure Measurement Time on cybersickness. A regression analysis was then used to characterize what might be driving any differences. Several steps were taken to determine which of the pool of candidate predictive variables (i.e., previous VR and gaming experience, FOV, IPD, field dependence, postural stability, female hormonal cycle, state/trait anxiety, migraine susceptibility, ethnicity, aerobic fitness, body mass index, physiological mechanisms) should be included in the regression analysis. First a univariate ANOVA was performed to evaluate significant gender differences among the potential predictive variables. The selection criterion chosen was whether or not each possible predictor variable was significantly different between the genders; those variables that were significantly different (set at *p* < 0.27 for univariate analysis; the more traditional 0.05 level can fail to identify important variables; Bursac et al., 2008) between the genders were included in the regression analysis. Next, a zero-order correlation analysis determined the strength of linear association among the predictor variables, as well as with the “recovery” SSQ TS metric. High correlation among predictor variables suggests redundant variable inclusion.

To further increase the predictability of the variables, especially given that an appropriate predictor to sample size ratio is 1:15, independent variables with the highest zero-correlation with the recovery SSQ TS metric were included first in the model. All categorical variables were dummy coded with males who fit the VR headset as the comparator. The IPD Fit metric classified males and females as out of and below the IPD range of the headset (<60.5 mm), within the range (60.5–74.4 mm), or out of and above the range (>74.4 mm). A binary classification of IPD Fit was then determined, which signified participants in IPD range for the HTC Vive or out of range (either below or above). The regression coefficient (β) of each predictor variable on recovery SSQ TS was calculated using SPSS version 24 multiple linear regression analysis. Models were evaluated for significant R2 change using an F-test and an a priori α level of 0.05, as well as multicollinearity using ≥0.20 as a cut off for tolerance and a variance inflation factor cutoff of ≥ 4.

### Results

**Figure 2:**
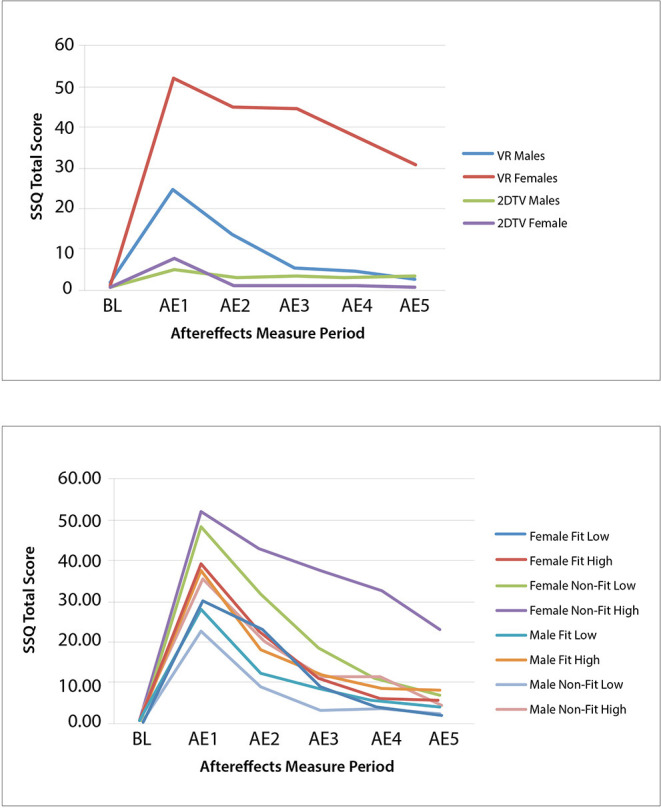
Experiment 1 **(Top)** and Experiment 2 **(Bottom)** group mean SSQ total score at baseline (BL) and each aftereffects (AE) measurement period for IPD fit and non–fit and motion sickness history low and high.

The results revealed that there were significant differences in the cybersickness experienced between the flatscreen TV and VR conditions. While there was a main effect of Gender [*F*_(1, 42)_ = 4.13, *p* < 0.049], and a main effect of Display Type [*F*_(1, 42)_ = 8.29, *p* < 0.006], there was also a significant interaction between Gender and Display Type [*F*_(1, 42)_ = 4.85, *p* < 0.033]; with Gender differences found for the VR display but not flatscreen TV. As expected, for both genders low levels of cybersickness were experienced with exposure to flatscreen TV immediately after exposure (female AE1 SSQ TS mean = 7.95; S.D. = 18.15; male AE1 SSQ TS mean = 5.61; S.D. = 8.48; see Table 4) and these low levels continued throughout the post exposure measurement periods (female AE5 SSQ TS mean = 0.94; S.D. = 2.64; male AE5 SSQ TS mean = 3.74; S.D. = 4.90; see Table 4). On the other hand, VR exposure proved problematic to both genders, but with some clear differences (see Table 4 and Figure 2-Top). Specifically, immediately after VR exposure the adverse effects in females (AE1 SSQ TS 52.11; S.D. = 41.63) were, on average, more than 2x that of males (SSQ TS mean = 24.93; S.D. = 32.69); this difference was significant [*F*_(1, 28)_ = 4.104, *p* = 0.05]. Further, these adverse effects persisted long after VR exposure for females (AE5 SSQ TS mean = 31.42; S.D. = 40.65), while males recovered much more quickly (AE5 SSQ TS mean = 3.24; S.D. = 6.76); this difference was significant [*F*_(1, 28)_ = 7.27, *p* = 0.012]. Females in the VR condition, on average, never returned to BL levels (see Table 4 and Figure 2-Top, AE5), while males, on average, recovered to BL within 30 min post exposure (see Figure 2-Top, AE3). A regression analysis was conducted to characterize these gender differences and identify which predictor variables may be driving them.

**Table 4:** Experiment 1 SSQ total score values at baseline (BL), immediately following exposure (aftereffect; AE1), and 1 h post exposure (aftereffect; AE5).

**Device**	**Gender**	**BL- Mean (SD)**	**AE1-Mean (SD)**	**AE5-Mean (SD)**
VR (HTC Vive)	Female	2.24 (3.69)	52.11 (41.63)^*^	31.42 (40.65)^*^
VR (HTC Vive)	Male	2.99 (3.22)	24.93 (32.69)^*^	3.24 (6.76)
Flatscreen TV	Female	1.40 (1.94)	7.95 (18.15)	0.94 (2.64)
Flatscreen TV	Male	1.87 (2.83)	5.61 (8.48)	3.74 (4.90)

* *p < 0.05 (different from BL)*.

Table 3 summarizes the results from the ANOVA analysis for identifying variables significantly contributing to gender differences. Based on these results, the variables targeted for inclusion in the regression analysis were VR and gaming experience, FOV, IPD Fit, field dependence, female hormonal cycle, state anxiety, ethnicity, aerobic fitness, and past motion sickness history, as each of these variables demonstrated significant differences between genders (see Table 3). The variables excluded from the regression analysis were postural stability, trait anxiety, migraine susceptibility, body mass index, and physiological mechanisms, all of which were not significantly different between the genders (see Table 3).

As shown in Table 5, FOV was highly correlated with the IPD Fit measure, so FOV was removed from the model, as IPD Fit was correlated with Recovery SSQ TS but FOV was not. As well, Hormonal Cycle was highly correlated with Gender, so Hormonal Cycle was removed from the model, as Gender was correlated with Recovery SSQ TS but Hormonal Cycle was not. All other predictor variables targeted for inclusion were systematically added and removed from the model based on the F-statistic and multicollinearity until the model could no longer be significantly improved.

**Table 5:** Experiment 1 predictor variable correlations.

	**Gender**	**VR experience**	**Gaming experience**	**FOV**	**IPD fit**	**Field dependence**	**Hormonal cycle**	**State anxiety**	**Ethnicity**	**Aerobic fitness**	**Motion sickness history**
Gender											
VR experience	−0.266										
Gaming experience	−0.208	0.113									
FOV	−0.221	0.002	0.070								
IPD fit	0.447^*^	−0.127	−0.040	−0.589^*^							
Field dependence	−0.121	0.299	0.281	−0.045	0.047						
Hormonal cycle	0.894^*^	−0.242	−0.110	−0.115	0.320	−0.117					
State anxiety	−0.133	−0.103	0.131	−0.127	−0.016	−0.126	−0.056				
Ethnicity	0.067	0.180	−0.088	−0.165	0.211	0.287	0.060	−0.300			
Aerobic fitness	−0.387	−0.043	−0.022	−0.013	−0.046	0.220	−0.389^*^	0.256	0.124		
Motion sick history	0.282	−0.198	−0.123	−0.124	0.378^*^	−0.079	0.162	0.307	0.062	0.415^*^	
SSQ (Dependent)	0.475^*^	−0.144	0.074	−0.045	0.489^*^	−0.113	0.332	−0.085	0.311	−0.175	0.453^*^

* *p < 0.05*.

Table 6 shows the results from the multiple linear regression analysis. Of the 30 participants in the VR headset condition, 26 participants had complete data for the regression analysis. The results show that IPD Fit was a strong and significant (*p* = 0.009) predictor of cybersickness. Past motion sickness history also contributed to the explanatory power of the model. The resulting model, which had an R2 = 0.469, Adjusted R2 = 0.420, RMSE = 27.77, *F*_(2, 23)_ = 9.70, *p* = 0.001, was as follows:

$$\begin{array}{l} E(Recovery\;SSQ\;TS)=-2.51+47.45^{*}IPD\;Fit\\ \;+4.66^{*}Motion\;Sickness\;History \end{array}$$

This model suggests that IPD non-fit and motion sickness history are positively correlated with cybersickness, with IPD non-fit being the most influential variable. This model accounted for 42.0% of the variability in cybersickness. Follow-up analyses indicated that the model passed the assumptions of multiple regression including normality and independence of residuals.

**Table 6:** Summary of multiple linear regression model of cybersickness for Experiment 1.

**Predictor variables in cybersickness model**	**β**	**SE**	**t**	**P > |t|**
(Intercept)	−2.51	8.38	−0.30	0.767
IPD Fit	47.45	16.63	2.85	0.009^*^
Past motion sickness history (MHQ)	4.66	2.62	1.78	0.103
Gender	0.250		1.47	0.156
VR experience	−0.012		−0.07	0.391
Gaming experience	0.137		0.877	0.941
Field dependence	−0.081		−0.490	0.629
State anxiety	−0.120		−0.722	0.473
Ethnicity	0.252		1.67	0.109
Aerobic fitness	−0.302		−1.90	0.072

* *p < 0.05*.

### Experiment 1 Summary

The primary finding from Experiment 1 is that the most significant driver of gender differences in cybersickness was IPD non-fit, with motion sickness history also contributing. The IPD differences found in the sample population under evaluation in this study are summarized in Table 7. The table includes the number of individuals in each condition for which the HTC Vive IPD adjustable range could not be fit to the participant's IPD. The average male IPD (mean = 65.33; S.D. = 2.99) was 4.1% wider than females (mean = 62.63; S.D. = 3.52) and this difference was significant [*F*_(1, 28)_ = 5.13, *p* = 0.031]. Within the female group, 5 of 15 or 33.3% (in line with expectations based on the US Army Anthropomorphic Survey [ANSUR] database; Gordon et al., 2014; see Table 2) of the females had an IPD that could not be properly fit to the VR headset, while all of the males fit. Of the five females whose IPD could not be fit, one had a low motion sickness history (MHQ ≤ 2). This individual had low sickness immediate post VR exposure (AE1 SSQ TS = 14.96) and recovered completely within 1 h post-VR exposure (AE5 SSQ TS = 0). The other four IPD non-fit females had a high motion sickness history (MHQ > 2) and these four females were profoundly sick immediate post VR exposure (AE1 SSQ TS mean = 74.8; S.D. = 48.76) and were not able to recover by AE5 (SSQ TS mean = 67.32; S.D. = 55.05). As all males could fit their IPD to the headset, no effects of IPD non-fit could be assessed for males. These results suggest that those for which a VR headset cannot be fit to their IPD and who have a high motion sickness history will be the most susceptible to cybersickness.

**Table 7:** Experiment 1 interpupillary distance differences between the genders.

**Gender**	**Mean**	**S.D**.	**Min**	**Max**	**HTC vive IPD Non-fit**	**F Stat**	***p*-value**
Females	62.63	3.52	57	70	5/15	5.13	0.031^*^
Males	65.33	2.99	61	71	0		

* *p < 0.05*.

Why would IPD non-fit drive higher levels of cybersickness. There are plenty of online blogs and developer sites that claim that a little bit of a blurred image in a VR headset due to a mismatched IPD is no problem (c.f. SteamVR, 2016, 2018). Yet, even if the IPD non-fit results in a small loss of visual acuity, this can have a substantial negative impact (Skrbek and Petrov, 2013). IPD non-fit can lead to increased fusional difficulty (Rolland and Hua, 2005), binocular stress, increased near point convergence, an esophoric (inward) shift in distance heterophoria, and a drop in visual acuity, as well as asthenopia (i.e., fatigue, eye pain, blurred vision, double vision, headache, general malaise, nausea; Mon-Williams et al., 1993; Regan and Price, 1993; Best, 1996). These adverse effects occur because IPD non-fit leads to misalignment of the VR headset optics and/or inappropriate binocular overlap, resulting in perceptual issues. Regan and Price (1993) found that only those with an IPD less than the interocular distance (IOD), which refers to the distance between the optical centers of the lens systems installed in the VR headset, experienced such visual discomfort, with the greater the mismatch between the two measures (IPD and IOD) resulting in greater reported side-effects.

In this study, the IOD or distance between the HTC Vive lenses was set to coincide with the participant's IPD whenever possible. This alignment is anticipated to mitigate misalignment between optics of the eyes and that of the VR headset. However, as researchers note, when an alignment cannot be achieved this will result in viewing the VR headset lens system on an off-center axis, which will in turn lead to prismatic distortions that drive eyestrain and visual discomfort (Regan and Price, 1993; Costello, 1997; Peli, 1999; Lee et al., 2008). It is thus not surprising that in the current study, females experienced higher levels and longer lasting cybersickness than males, as one third of females had a smaller IPD than the VR headset. This mismatch between the IOD and IPD for woman is not correctible in software as it is a hardware issue and may lead to a higher likelihood over males of experiencing the taxing effects of a divergence demand such as visual fatigue (Costello, 1997).

Theoretical research suggests that the mismatch between inter-screen distance (ISD) and IOD is a driver of accommodation-convergence issues (Howarth, 1999). Choosing the correct eye-point for rendering computer generated graphics potentially diminishes these negative effects, especially depth errors, since choosing the correct eye-point will account for near- or far- field headset screen settings and aligns the center of the display with the optics and the correct eye-point of the end-user (Rolland et al., 2004). By adjusting the IPD of the end user and setting the IPD in the system software, the displays can be aligned to the eye-point of the participant if the headset IPD adjustable range allows. However, because the VR headset did not represent the full range of IPDs of the participants (i.e., while all males could properly fit their IPD to the headset, a third of the females could not be properly fit), there is a potential that the negative effects reported could be due to other types of interactions between the technology and rendered images.

If IPD non-fit is the main driver of gender differences in cybersickness, then females whose IPD fit the VR headset should experience cybersickness in a manner similar to males. Specifically, they should experience cybersickness at comparable levels upon immediate post VR exposure, and then they should recover at a rate similar to males. To test these assumptions, a second experiment was run.

## Experiment 2

### Materials and Methods

The purpose of Experiment 2 was to determine if females whose IPD could be fit to the VR headset experienced cybersickness in a manner similar to males. Based on the results of Experiment 1, it was anticipated that females would experience higher levels and longer lasting cybersickness than males only when their IPD could not be fit to the headset; and potentially only when their IPD was smaller than the IOD. It was also expected that both females and males with high motion sickness histories would experience cybersickness at higher levels as compared to those with low histories.

#### Participants

Adults aged 18–30 years, balanced between genders participated in this study. Participants were recruited through a market research firm. A total of 120 participants were recruited for the study based on their fit to one of eight experimental groups, which were defined according to gender (male vs. female), IPD (fit vs. non-fit), and motion sickness history (low vs. high). MHQ was defined as follows: Low Motion Sickness History = MHQ < = 2; High Motion Sickness History = MHQ > 2. This research complied with the American Psychological Association Code of Ethics and was approved by the Institutional Review Board at Copernicus Group. Informed consent was obtained from each participant and all participants were compensated for their time in the experiment. Data from the 30 VR participants from Experiment 1 were also included in the Experiment 2 data analysis, and the IPD Fit/Non-Fit and MHQ Low/High were identified for each Experiment 1 VR participant.

#### Experimental Design

The experiment was a mixed design, with 2 (gender) × 2 (VR headset IPD fit type) × 2 (motion sickness history type) between factors and a 5 (post exposure measurement time) within factor. Gender types were either male or female. VR headset IPD fit type was either IPD Fit or IPD Non-Fit. Motion sickness history type was either Low or High. The post exposure measurement times were 0, 15, 30, 45, and 60 min.

Beyond the randomization of participants to groups, the Equipment and Display Content, Procedure, Dependent Measures, and Data Analysis were the same as in Experiment 1. One additional Predictor Variable was added to Experiment 2, which was Exposure Duration. This was added to address any potential differences in drop-out rates.

### Results

Complete datasets from 117 of the 120 participants in Experiment 2 were obtained and combined with the 30 VR participants from Experiment 1 to run the ANOVA, providing a total sample size of 147 participants. The combined data led to a total of: 40 female IPD Fit participants (19 were low MHQ; 21 were high MHQ), 45 male IPD Fit participants (25 were low MHQ; 20 were high MHQ), 34 female IPD Non-Fit participants (15 were low MHQ; 19 were high MHQ), and 28 male IPD Non-Fit participants (15 were low MHQ; 13 were high MHQ). Thus, when combining Experiment 1 and 2 data, there were a total of 85 IPD Fit VR participants and 62 IPD Non-Fit VR participants. The mixed-model ANOVA results revealed significant main effects for Gender [*F*_(1, 139)_ = 7.36, *p* = 0.008] and MHQ, [*F*_(1, 139)_ = 5.40, *p* = 0.022], as well as a significant interaction of Gender × MHQ × IPD Fit [*F*_(1, 139)_ = 4.24, *p* = 0.008]. The results revealed that, as expected, females whose IPD fit the VR headset experienced cybersickness in a manner similar to males (see Table 8 and Figure 2-Bottom). Specifically, immediately after VR exposure, the average Recovery SSQ TS levels for those in the IPD Fit condition were high in both those with a high motion sickness history (females: AE1 SSQ TS mean = 40.25; S.D. = 35.99; males: AE1 SSQ TS mean = 32.91; S.D. = 36.78) and those with a low motion sickness history (females: SSQ TS mean = 28.94; S.D. = 30.25; males: AE1 SSQ TS mean = 23.49; S.D. = 28.98). There were no significant gender differences in the IPD Fit groups at AE1 (*n* = 85), regardless of motion sickness history [*F*_(3, 81)_ = 1.029, *p* = 0.384]. All those who could fit their IPD to the VR headset recovered within 1 h post VR exposure, regardless of motion sickness history (high motion sickness history females: AE5 SSQ TS mean = 7.48; S.D. = 11.47; males: AE5 SSQ TS mean = 5.98; S.D. = 13.64; low motion sickness history females: AE5 SSQ TS mean = 4.53; S.D. = 19.13; males: AE5 SSQ TS mean = 0.45; S.D. = 5.10. There were no significant gender differences in the IPD Fit groups at AE5 (*n* = 85), regardless of motion sickness history [*F*_(3, 81)_ = 1.29, *p* = 0.283]; further these groups had no significant differences from BL at AE5 [*t*_(84)_ = −1.656, *p* = 0.104].

**Table 8:** Experiment 2 self-reported SSQ total score values at baseline (BL), immediately following exposure (aftereffect; AE1), and 1 h post exposure (aftereffect; AE5).

**Gender**	**IPD**	**Motion sickness history**	**BL- Mean (SD)**	**AE1-Mean (SD)**	**AE5-Mean (SD)**
Female	Fit	Low	1.77(2.89)	28.94(30.25)^*^	4.53 (19.13)
		High	1.96 (3.26)	40.25 (35.99)^*^	7.48 (11.47)
	Non-Fit	Low	1.50 (2.76)	46.87(50.17)^*^	4.99 (9.76)
		High	2.76 (3.26)	53.54 (41.08)^*^	29.53 (48.31)^*^
Male	Fit	Low	2.09 (2.87)	23.49 (28.98)^*^	0.45 (5.10)
		High	2.06 (3.09)	32.91 (36.78)^*^	5.98 (13.64)
	Non-Fit	Low	0.50 (1.32)	22.19 (22.18)^*^	1.99 (5.45)
		High	3.16 (3.69)	31.65 (32.63)^*^	0.86 (7.19)

* *p < 0.05 (different from BL)*.

Immediately after VR exposure, the adverse effects in those that had an IPD non-fit were, on average, high in both those with a high motion sickness history (female AE1 SSQ TS mean = 53.54; S.D. = 41.08; male AE1 SSQ TS mean = 31.65; S.D. = 32.63) and those with a low motion sickness history (females AE1 SSQ TS mean = 46.87; S.D. = 50.17; males AE1 SSQ TS mean = 22.19; S.D. = 22.18). There were no significant gender effects in the IPD Non-Fit groups at AE1 (*n* = 62), regardless of motion sickness history [*F*_(3, 58)_ = 2.25, *p* = 0.092]. There was a statistically significant difference in AE5 SSQ TS among the groups [*F*_(3, 58)_ = 4.19, *p* = 0.009; *n* = 62]. A Tukey *post-hoc* analysis showed that these adverse aftereffects persisted long after VR exposure only for those females with an IPD non-fit and high motion sickness history (females AE5 SSQ TS mean = 29.53; S.D. = 48.31) as compared to females with low motion sickness history (AE5 SSQ TS mean = 4.99, S.D. = 9.76, *p* = 0.047), males with low motion sickness history (AE5 SSQ TS mean = 1.99, S.D. = 5.45, *p* = 0.033), or males with high motion sickness history (AE5 SSQ TS mean = 0.86, S.D. = 7.19, *p* = 0.034). Both females (female AE5 SSQ TS mean = 4.53; S.D. = 19.13) and males (male AE5 SSQ TS mean = 1.99; S.D. = 5.45) with an IPD non-fit and low motion sickness history recovered by the final measurement period. Females in the IPD Non-Fit, High Motion Sickness History condition also had, on average, less VR exposure duration (i.e., tended to drop-out; Mean Exposure Duration = 15.95 min; S.D. = 6.42) as compared to both males (Mean Exposure Duration = 20 min; S.D. = 0.00, i.e., no dropouts) and females (Mean Exposure Duration = 19.81 min; S.D. = 0.544) in the IPD Non-Fit, Low Motion Sickness History conditions; this difference was significant [*F*_(7, 139)_ = 2.71, *p* = 0.012].

Table 9 shows the results from the multiple linear regression analysis from Experiment 2. Of the 120 participants in Experiment 2, 109 participants had complete data for the regression analysis. These data were combined with the 26 participants from Experiment 1 with complete data sets for the regression analysis, providing 135 complete data sets. The first step in the analysis was the same, the univariate analysis of each possible predictor variable, with the results mostly replicating the Experiment 1 findings (i.e., VR and gaming experience, FOV, IPD fit, female hormonal cycle, state anxiety, ethnicity, aerobic fitness, and past motion sickness history demonstrated significant differences between genders and were targeted for inclusion in the regression analysis), with the addition of EGG (bradygastria) and exposure duration also demonstrating significant gender differences (see Table 3) and thus these two additional variables were targeted for regression analysis inclusion. The variables excluded from the regression analysis were postural stability, trait anxiety, migraine susceptibility, and body mass index, which were the same as Experiment 1, with the addition of field dependence, all of which were not significantly different between the genders (see Table 3).

**Table 9:** Experiment 2 predictor variable correlations.

	**Gender**	**VR experience**	**Gaming experience**	**FOV**	**IPD fit**	**Hormonal cycle**	**State anxiety**	**Ethnicity**	**Aerobic fitness**	**Motion sickness history**	**Exposure duration**	**EGG bradygastria**
Gender												
VR experience	−0.133											
Gaming experience	−0.208^*^	0.168^*^										
FOV	−0.127	−0.027	0.127									
IPD fit	0.083	0.034	0.028	−0.147								
Hormonal cycle	0.811^*^	−0.117	−0.214^*^	−0.048	0.061							
State anxiety	−0.061	−0.062	−0.016	−0.098	−0.005	−0.096						
Ethnicity	−0.060	0.051	−0.089	0.035	−0.027	0.039	−0.108					
Aerobic fitness	−0.521^*^	−0.092	0.057	0.294^*^	−0.103	−0.413^*^	−0.002	0.167^*^				
Motion sickness history	0.169^*^	−0.138	−0.122	−0.147	0.115	0.076	0.164^*^	−0.025	−0.021			
Exposure duration	−0.173^*^	0.039	0.005	0.021	0.012	−0.086	−0.150	0.091	0.072	−0.177^*^		
EGG bradygastria	0.199^*^	0.007	0.068	−0.050	0.031	0.196^*^	−0.067	0.081	−0.073	0.041	0.098	
SSQ (Dependent)	0.211^*^	−0.048	0.095	0.014	0.192^*^	0.141	0.061	0.001	−0.032	0.360^*^	−0.365^*^	0.230^*^

* *p < 0.05*.

As in Experiment 1, in Experiment 2 Hormonal Cycle had a very high linear association with Gender, so Hormonal Cycle was removed from the model, as Gender was correlated with Recovery SSQ TS but Hormonal Cycle was not (see Table 9). All other predictor variables targeted for inclusion were systematically added and removed from the model based on the F-statistic and multicollinearity until the model could no longer be significantly improved.

Table 10 shows the results from the multiple linear regression analysis. The resulting model, which had an R2 = 0.322, Adjusted R2 = 0.301, RMSE = 20.06, *F*_(4, 130)_ = 15.40, *p* = 0.001, was as follows:

$$\begin{array}{l} E(Recovery\;SSQ\;TS)=28.44+8.22^{*}IPD\;Fit\\ \;+2.62^{*}Motion\;Sickness\;History\\ \;+.421^{*}EGG\;Bradygastria\\ \;-2.20^{*}Exposure\;Duration \end{array}$$

This model suggests that IPD non-fit, motion sickness history, and bradygastria are positively correlated with cybersickness, while exposure duration (i.e., how long an individual was able to remain in VR) is negatively correlated to cybersickness. As in Experiment 1, IPD non-fit was found to be the most influential variable, followed by motion sickness history. This model accounted for 32.2% of the variability in cybersickness. Follow-up analyses indicated that the model passed the assumptions of multiple regression including normality and independence of residuals.

**Table 10:** Summary of multiple linear regression model of cybersickness for Experiment 1 and Experiment 2 combined data.

**Predictor variables in cybersickness model**	**β**	**SE**	**t**	**P > |t|**
(Intercept)	28.44	10.52	2.70	0.008^*^
IPD fit	8.22	3.53	2.32	0.022^*^
Past motion sickness history (MHQ)	2.62	0.696	3.763	0.000^*^
Exposure duration	−2.20	0.511	−4.30	0.000^*^
EGG bradygastria	0.421	0.120	3.51	0.001^*^
Gender	0.065		0.845	0.400
VR exposure	−0.022		−0.293	0.770
Gaming experience	0.122		1.66	0.098
Field of view	0.081		1.10	0.271
Female hormonal cycle	0.044		0.589	0.557
State anxiety	−0.004		−0.050	0.960
Ethnicity	−0.006		−0.084	0.933
Aerobic fitness	0.032		0.410	0.682

* *p < 0.05*.

### Experiment 2 Summary

**Figure 3:**
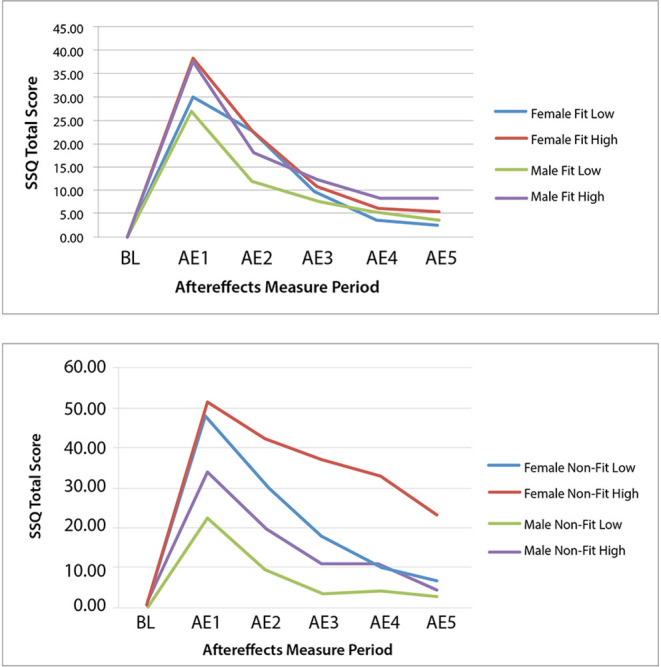
Experiment 2 group mean SSQ total score at baseline (BL) and each aftereffects (AE) measurement period for the IPD fit **(Top)** and non–fit **(Bottom)** and motion sickness history low and high experimental groups.

Similar to Experiment 1, Experiment 2 found that the primary driver of cybersickness is IPD non-fit, followed by motion sickness history. Experiment 2 also found higher EGG (bradygastria) and higher dropout rates (i.e., lower exposure duration) associated with higher levels of cybersickness. In terms of EGG, previous research has indicated that bradygastria is a correlate of motion sickness (Lang et al., 1999) and changes to bradygastria immediately precede nausea (Kim et al., 2005; Dennison et al., 2016); this associated objective physiological response, in effect, validates the subjective SSQ TS results in the current study. In terms of exposure duration, increased cybersickness has been previously associated with higher drop-out rates (Stanney et al., 1999), and the negative correlation for exposure duration mirrors this finding. Further, the results from Experiment 2 demonstrated that females whose IPD could be fit to the VR headset experienced cybersickness in a manner similar to males, while those females who could not be fit experienced more severe and more persistent cybersickness. For females and males whose IPD could be fit to the VR headset, they experienced high levels of cybersickness immediately after VR exposure but fully recovered within 1 h post exposure, regardless of motion sickness history (all AE5 SSQ TS not significantly different than BL; see Table 8 and Figure 3-Top).

In the high motion sickness history conditions, IPD non-fit did not affect males to the same degree as females, as males were able to recover to baseline while females were not (see Table 8 and Figure 3-Bottom). This may be because males' IPD non-fit was not as severe as females' and a greater degree of mismatch has been associated with more severe adverse effects (Mon-Williams et al., 1993; Regan and Price, 1993; Best, 1996). In general, all but one female in the IPD Non-Fit condition had an IPD smaller than the adjustable IPD range, and the average IPD of this group was 57.52 mm (S.D. = 1.77). All but two males in the IPD Non-Fit condition had an IPD smaller than the adjustable IPD range, and the average IPD of this group was 58.87 mm (S.D. = 1.14). There was a significant difference [*F*_(1, 60)_ = 7.48, *p* = 0.008], in the severity of the IPD non-fit between males and females (*n* = 62), with females having a more severe non-fit. This greater degree of IPD non-fit was associated with a significantly higher level of cybersickness for females vs. males immediately following VR exposure (see Tables 5, 8 and Figures 2, 3).

## General Discussion

This study sought to identify the main drivers of gender differences associated with the adverse effects of VR exposure. Two experiments were conducted, the first to investigate the many variables that could contribute to gender differences and the second to validate and further explore the findings of the first. In both experiments, IPD non-fit was found to be the main driver of cybersickness, with motion sickness history a secondary driver.

### Interpupillary Distance

Quite interestingly, it was not an inherent characteristic of females but rather a characteristic of the VR headset itself, IPD non-fit, that was found to be the primary driver of cybersickness in both experiments. To properly view objects in a virtual environment, most VR headsets have a variable IPD range that allows an individual to align the center of their pupils with the center of the VR lenses. Any deviation between IPD and IOD can cause a host of visual issues, as well as asthenopia (Mon-Williams et al., 1993; Regan and Price, 1993; Best, 1996; Rolland and Hua, 2005). To resolve this issue and allow both females and males to be able to properly center their pupils to the lenses, the IPD range needs to be adjustable from ~ 50 to 77 mm (Dodgson, 2004; Gordon et al., 2014). As can be seen in Table 2, the Sony PlayStation headset accommodates this range but many other VR headsets on the market today do not (e.g., Samsung Gear VR, Oculus Rift, Oculus Rift S, Oculus Quest, HTC Vive, HTC Vive Pro). Another option is to custom fit VR headsets to an individual, much like eyeglasses (Luckey, 2019). Software IPD adjustment helps with scale issues but does not address issues with hard to fuse imagery, blurry images, distortion, or VOR mismatches (Luckey, 2019), thus it may not be the panacea many believe it to be. Other possible alternatives to resolve this issue include gaze-contingent and adaptive focus displays (Padmanaban et al., 2017), yet these solutions still pose challenges to the human visual system (Rolland et al., 2000; Mercier et al., 2017). Until such modifications to VR headsets are made, females will be at a particular disadvantage with regard to cybersickness because in general the IPD range in current VR headsets accommodates substantially fewer females as compared to males (see Table 2) and their IPD mismatch will likely be more severe than males.

Beyond widening the IPD range, there are a number of design parameters that need to be considered to ensure the design of VR headsets better accommodates human physiology. Specifically, Robinett and Rolland (1992) noted that VR headsets are cross compared using engineering techniques that use a reduced eye model that sets average human constraints based on male specific measures, such as performing tests using a static IPD of 64 or 65 mm (note, Male mean IPD is 64.0 mm [S.D. = 3.4 mm]; Gordon et al., 2014). These model simplifications ignore performance limitations compared to the human eye (e.g., FOV, resolution). Specifically, the VR headset parameters of resolution, image focus, contrast, brightness, and frame rate are interdependent parameters of a VR headset that affect viewability of complex, dynamic VR imagery. At the same time, the human eye is an optical system that is functionally limited much like the VR headset in such parameters as display resolution and image quality. Clearly explicating these limitations and avoiding making display choices that do not match human visual capabilities (see potential mismatches in Table 11) will reduce cybersickness. By understanding these challenges, VR headset design can be much improved to better accommodate the human visual system.

**Table 11:** VR headset parameters compared to human visual system.

VR headset parameters^*^ (typical values)	**Human visual system** **(typical values)**
Monocular field of view: 20–65° Binocular field of view: 20–110°	Monocular field of view: 160° horizontal Binocular field of view: 200° horizontal
Image quality: 2.0–4.1 arc min	Visual acuity: .8 arc min in fovea (20/20 Snellen)
Exit pupil: 12 mm	Pupil: 2–10 mm depends on lighting conditions
Luminance range: 50–1,000 cd/m^2^	Brightness: Scoptic 10^−6^ to 3 cd/m^2^ Photopic > 3 cd/m^2^
Image focus: Fixed	Accommodation: Dynamic and Cornea + Lens have a combined power of 58.6 Diopters
Eye relief (ER): 15–50 mm	Eyeglasses imposed ER ≥ 17 mm
Optical separation between L and R lenses, 58–72mm, widest range available^**^ based on IPD of user	Interpupillary eye distance (IPD): distance between the two eyes, 50–77 mm for 95% of Asians, Caucasians, and African Americans

* *VR headset parameters adapted from Rolland and Hua (2005) and Cakmakci and Rolland (2006)*.

** *see Table 2*.

**Figure 4:**
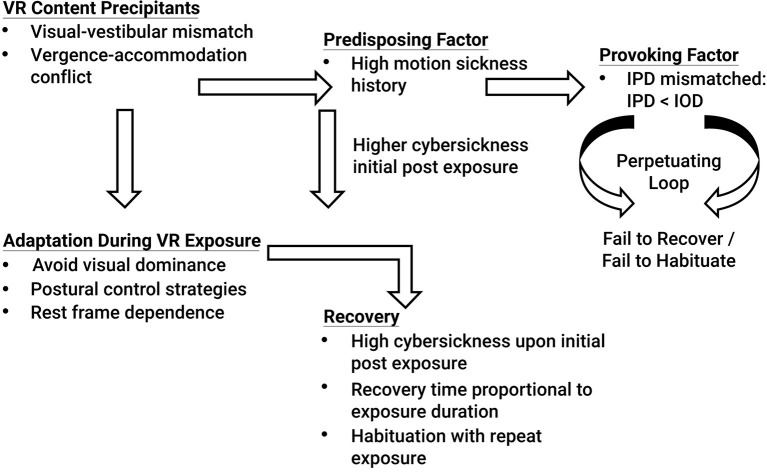
Mechanisms of cybersickness.

The results of Experiment 2 suggest that if an individual's IPD can be properly fit to the VR headset, gender differences in cybersickness are not expected (see Table 8 and Figure 3-Top). Cybersickness is still expected, as was experienced in Experiment 2 (see AE1 in Table 8) due to visual-vestibular mismatches (Reason and Brand, 1975; Oman, 1998) and vergence-accommodation conflict (Szpak et al., 2019). Specifically, if designers create content with a great deal of vection (Webb and Griffin, 2003) associated with high levels of visual-vestibular mismatches and/or content with a large conflict between vergence and focal distances (Hoffman et al., 2008), these conflicts are expected to precipitate sickness (see Figure 4). Based on the results of Experiment 2, sickness levels upon immediate post VR exposure are expected to be higher in those with a high motion sickness history. However, regardless of motion sickness history, these adverse effects are expected to dissipate once adaptations (e.g., avoiding visual dominance, adopting postural control strategies such as through active viewpoint control, and cuing off a rest frame to minimize visual-vestibular mismatches) and habituation with repeat exposures kick-in (see Figure 4), as was experienced in Experiment 2 (see AE5 in Table 8), and in proportion to exposure duration for those individuals that can properly fit their IPD (Kennedy et al., 2000; Murata, 2004). Note that the VR content used in this study was designed to induce cybersickness by creating content that was intended to be provocative. Yet, even with potent VR content, the results from Experiment 2 demonstrated that when the IPD could be properly fit to the VR headset, both males and females recovered from the adverse effects of VR exposure within 1 h post VR exposure, regardless of motion sickness history (see Figure 3-Bottom). It is only when an individual has the provoking factor of an IPD that cannot be properly fit, specifically when the IPD of an individual is smaller than the IOD, and the individual has the predisposing factor of a high motion sickness history that the individual is expected to enter a perpetuating loop that does not allow cybersickness recovery and habituation (see Figure 4).

### Motion Sickness History

Many individuals experience motion sensitivity during activities such as reading when being a passenger in an automobile (Turner and Griffin, 1999), riding on a boat (Lawther and Griffin, 1986; 1988; Cooper et al., 1997), riding on a train (Kaplan, 1964), and flying (Lederer and Kidera, 1954; Turner et al., 2000), with these activities leading to feelings of dizziness, general malaise, nausea, blurry vision, and other such adverse effects. Individuals with such a history of motion sensitivity may be more susceptible to cybersickness in virtual environment than those without such history. Motion sensitivity has been suggested to be caused by vestibular dysfunction (Akin and Davenport, 2003) and/or an over-reliance on the visual system with a residual deficit of the vestibular system (Akiduki et al., 2003). Daily, short (5 min) vestibular adaptation exercises in those with vestibular dysfunction have been shown to be effective in reducing symptomatology (Alyahya et al., 2016). In fact, habituation (i.e., desensitization with repeat exposures) has been suggested to be the most effective countermeasure to motion sickness, even more so than anti-motion sickness drugs (Cowings and Toscano, 2000). Specifically, over repeat exposures to VR environments, habituation may occur in which symptomatology decreases (Kennedy and Graybiel, 1965; Biocca, 1992; McCauley and Sharkey, 1992; Regan, 1995; Domeyer et al., 2013; Welch, 2014). Habituation is oftentimes highly effective (Golding, 2017), perhaps as high as 85% effective (Benson, 1999). Habituation protocols would involve designing VR applications with stepwise increments in stimulus intensity coupled with frequent exposures of slowly increasing duration, which may allow motion sensitive individuals to acclimate to the experience, enable initial faster recovery and more sessions to be tolerated. Based on Welch (2014), important elements of a habituation protocol would include: (1) active (not passive) interaction within the VR environment, coupled with the visual consequences associated with these actions (reafference), (2) immediate feedback to this interaction (any transport delays, response lags, etc. will hinder adaptation, however, if these lags are consistent, then adaptation may still be achieved), (3) incremental (rather than massed) exposure, with progressive VR stimulus strength (e.g., start with mild, slow movements, constant velocity, etc.), and (4) the use of distributed practice (e.g., 2–5 day intersession intervals). However, based on the results of these studies, should the VR headset pose an IPD non-fit, such habituation protocols may not prove effective.

If IPD fit is achieved, such habituation protocols hold great promise in addressing gender differences, as females are generally more disposed, as compared to males, to benefit from such conditioning countermeasures (Rohleder et al., 2006; Stockhorst et al., 2007). For example, Rohleder et al. (2006) demonstrated that physiological habituation to a repetitive rotation experience was demonstrated only in females via habituation of a rotation-induced cortisol response, whereas males continued to show cortisol sensitivity. Thus, even though females oftentimes report being more highly susceptible to motion sickness than males (Lentz and Collins, 1977; Park and Hu, 1999; Dobie et al., 2001; Graeber and Stanney, 2002; Stanney et al., 2003; Wilson and Kinsela, 2017), and this susceptibility may lead to higher levels of cybersickness in virtual environments, susceptibility difference for both females and males can be counteracted via appropriate habituation practices.

It is also interesting to note that when IPD was properly fit, even those with a high motion sickness history could recover within 1 h post exposure (see Figure 3-Top). Thus, the impact of motion sickness history is not as profound as that of IPD non-fit, which the regression model confirmed.

### Limitations

Given the vast range of motion sensitivity in the general population, which varies by about 10-1 (Lackner, 2014), a larger sample would have been desirable. Further, while the SSQ (Kennedy et al., 1993) is a standard measure of motion sickness that has been used for decades (Bulk et al., 2013), future research should add objective measures of the adverse aftereffects of VR exposure to confirm subjective reports of cybersickness, e.g., measures of ataxia, VOR shift, kinesthetic position sense shift (Kennedy et al., 1998).

## Conclusions

In summary, Experiment 1 identified that IPD non-fit is a primary driver of gender differences in cybersickness. Experiment 2 confirmed this finding and further demonstrated that when an individual's IPD could be properly fit to the VR headset, females experienced cybersickness in a manner similar to males, with high levels immediately post VR exposure and recovery within 1 h post exposure following a 20 min provocative VR exposure. As more females were unable to properly fit their IPD to currently available VR headsets, and any IPD non-fit experienced was more extreme in females than males, VR technology was indeed found to be sexist, but it does not have to be. If VR headset manufacturers implement an IPD adjustable range of ~ 50 to 77 mm to capture >99% of both females and males, it is anticipated that a far greater number of females will be able to harness the performance enhancing potential of VR technology. In addition, motion sickness susceptibility contributes to higher levels of cybersickness and this can be counteracted via habituation protocols.

## Data Availability Statement

The datasets generated for this study are available on request to the corresponding author.

## Ethics Statement

The studies involving human participants were reviewed and approved by Copernicus Group. The participants provided their written informed consent to participate in this study.

## Author Contributions

KS conducted the literature review, designed the experiments, directed the study, and was the lead author of the paper. CF led the data analytics. LF reviewed and provided feedback on all aspects of this research.

### Conflict of Interest

KS and CF are employed by Design Interactive, Inc. LF is employed by Lockheed Martin Corporate.
